# Systemic Immunomodulatory Effects of Full-Body Blue Light Therapy in Psoriasis Vulgaris Patients

**DOI:** 10.3390/jcm15135109

**Published:** 2026-07-01

**Authors:** Daniel Nolberczak, Aleksandra Lesiak, Magdalena Sadowska, Igor Aleksander Bednarski, Natalia Bień, Joanna Narbutt

**Affiliations:** 1 Department of Dermatology, Pediatric Dermatology and Dermatological Oncology, Medical University of Lodz, 92-215 Lodz, Poland; aleksandra.lesiak@umed.lodz.pl (A.L.); magdalena.sadowska.umed@gmail.com (M.S.);; 2 Laboratory of Autoinflammatory, Genetic and Rare Skin Disorders, Medical University of Lodz, 92-215 Lodz, Poland; 3 Department of Neurology, Central University Hospital, 92-213 Lodz, Poland

**Keywords:** blue light therapy, psoriasis vulgaris, kynurenine pathway

## Abstract

**Background/Objectives:** Psoriasis vulgaris is a chronic inflammatory skin condition with significant psychosocial burden. While phototherapy remains one of the most widely used treatment regimens, novel modalities like blue light therapy offer UV-free alternatives with potentially more favorable safety profiles, but their systemic immunomodulatory effects remain poorly understood. We aimed to evaluate the impact of full-body blue light irradiation on clinical outcomes and selected systemic biochemical and immunological markers in patients with mild-to-moderate psoriasis vulgaris. **Methods:** This preliminary study involved 21 patients (13 females, 8 males) with mild-to-moderate psoriasis vulgaris. Participants received ten sessions of full-body blue light therapy (453 nm, 40 mW/cm^2^, 30 min per session). Clinical assessments (PASI, PGA, DLQI, VAS, Pruritus Scale) and serum analyses of inflammatory (TNF-α, IL-13, IL-17, IL-31), metabolic (adiponectin, 25(OH)D3), and neuroimmune markers (serotonin, kynurenic acid, quinolinic acid) were performed pre- and post-treatment. **Results:** Significant improvements were observed in PASI, PGA, DLQI, and pruritus scores (*p* < 0.05). 25(OH)D3, serotonin, and kynurenic acid levels increased significantly, while IL-31 and IL-17 levels decreased and IL-13 levels increased; TNF-α, adiponectin, and quinolinic acid levels showed no significant changes. Counterintuitively, correlation analysis demonstrated a moderate positive association between changes in IL-13 and PASI improvement (r = 0.51, *p* = 0.02), while changes in other biochemical parameters were not significantly associated with clinical outcomes. **Conclusions:** Full-body blue light therapy resulted in significant clinical improvement accompanied by heterogeneous systemic immunometabolic changes. These findings suggest complex, pathway-specific immunomodulation, but this requires further investigation in larger controlled studies.

## 1. Introduction

Psoriasis vulgaris (PV) is one of the most prevalent chronic inflammatory skin disorders, affecting approximately 5% of the general population [1]. Beyond the visible lesions and physical discomfort, psoriasis significantly impacts patients’ psychosocial well-being and contributes to considerable healthcare costs, particularly in high-income countries [2]. Despite significant advancements in biologic therapies, phototherapy using ultraviolet A and B (UVA, UVB) remains a cornerstone in the management of psoriasis [3]. However, given the chronic nature of PV and the potential long-term risks associated with established treatments, there is a continued need for novel therapeutic strategies that combine effectiveness with improved safety and patient convenience.

In recent years, light-based therapies have become an integral component of dermatological treatment, encompassing ultraviolet phototherapy, laser systems, and visible light technologies. Advances in photomedicine have enabled the development of therapeutic approaches based on the interaction between specific wavelengths and cutaneous chromophores, allowing effective treatment while minimizing damage to surrounding tissues. The principle of selective photothermolysis has been particularly important for the treatment of pigmentary disorders, vascular lesions, and inflammatory skin diseases, and it has contributed to the expanding role of non-invasive light-based modalities in dermatology [4,5]. Within this context, blue light therapy has emerged as a promising UV-free, non-invasive option for managing various dermatological conditions, including psoriasis. Utilizing light-emitting diode (LED) technology, blue light (typically at 453 nm) has demonstrated anti-proliferative effects on keratinocytes and immunomodulatory properties, without the DNA-damaging risks inherent to UV-based therapies [6]. Preliminary studies suggest that blue light therapy may offer treatment efficacy comparable to conventional phototherapies in patients with mild-to-moderate psoriasis [7,8].

To fully optimize the therapeutic potential of blue light, a deeper understanding of its impact on the specific molecular mediators driving psoriatic inflammation is essential. The pathogenesis of psoriasis involves the dysregulation of numerous biomolecules, including cytokines central to the IL-23/Th17 axis (e.g., IL-17, IL-23, TNF-α) [8,9], immunomodulatory factors like vitamin D and adiponectin [10,11,12], the itch-mediating cytokine IL-31, and neuroimmune modulators such as serotonin and kynurenine pathway metabolites (kynurenic and quinolinic acid) [9,13]. While UV-free blue light therapy shows promise through local effects on keratinocyte homeostasis and broad immunomodulation [4,6], its direct influence on core psoriatic cytokines (TNF-α, IL-13, IL-17) and other key biomarkers like adiponectin within the specific context of psoriasis remains insufficiently elucidated. Thus, the aim of our study was to investigate the systemic effects of blue light therapy in patients with psoriasis by assessing changes in key biochemical and immunological markers, including 25-hydroxyvitamin D3, TNF-α, IL-13, IL-31, IL-17, adiponectin, serotonin, kynurenic acid, and quinolinic acid.

## 2. Materials and Methods

### 2.1. Study Group

The study included a total of 21 participants (8 males and 13 females) with mild-to-moderate psoriasis vulgaris not previously receiving biological treatment and other forms of phototherapy. The exclusion criteria were concomitant conditions/diseases with photosensitivity (porphyria, solar urticaria), immunosuppression, active or cured skin cancers, any current therapy that uses photosensitizing drugs, uncontrolled hypertension, having undergone myocardial infarction or stroke, and atherosclerosis. Pregnant and breastfeeding women were also excluded from the study. Any previous topical antipsoriatic treatment was discontinued at least 4 weeks before baseline, and no topical anti-inflammatory or antipsoriatic therapy was administered during the study period. Male participants had a mean age of 39.75 years, while females were slightly younger, with a mean age of 36.92 years. The average body weight for the whole group was 69.19 kg, with males weighing more on average (78.25 kg) compared to females (62.00 kg). Mean height was 168.62 cm overall, with males averaging 174.88 cm and females 164.77 cm. In terms of Fitzpatrick Skin Phototype, the majority of participants were type III (n = 13), while the remaining were type II (n = 8). Detailed baseline characteristics of the study group are shown in Table 1.

All participants underwent 10 full-body blue light irradiation using Phlecs Full Body Blue 1.0 (Phlecs, Eindhoven, The Netherlands) emitting 453 nm light (irradiance 40 mW/cm^2^) for 30 min a day for 5 consecutive days each week (2 days of pause). Before starting the study and after the full irradiation regimen, all participants were assessed using the Psoriasis Area and Severity Index (PASI), the Physician Global Assessment (PGA), the Dermatology Life Quality Index (DLQI), Visual Analog Scales (VASs) for pruritus and skin lesions, and the 10-Item Pruritus Severity Scale. Serum samples were taken from all individuals to measure the levels of immuno-inflammatory markers (TNF-α, IL-13, IL-17, IL-31), metabolic and endocrine markers (adiponectin, and 25-hydroxyvitamin D3), and neuroimmune and kynurenine pathway metabolites, such as serotonin (5-HT), quinolinic acid (QA) and kynurenic acid (KYNA). The approval of the Bioethical Commission (No. RNN/312/20/KE) was obtained before conducting the study. All participants signed written, informed consent before enrollment into the study.

### 2.2. Biological Material Collection

Blood samples were collected once from the antecubital vein using vacuum tubes: approximately 8 mL in clot-activator tubes and 4 mL in EDTA tubes. A closed collection system with sterile needles and an adapter was used. After clot retraction at room temperature, samples were centrifuged at 3000× *g* for 10 min (similarly for EDTA tubes). Serum and plasma were separated, aliquoted into Eppendorf tubes, and stored at −20 °C to −70 °C. Before analysis, samples were thawed and thoroughly mixed. No samples were refrozen.

### 2.3. Biochemical Analyses

Adiponectin concentration was quantified using the BioVendor Adiponectin ELISA (BioVendor, Brno, Czech Republic) (Competitive). Absorbance was read at 450 nm. Concentrations were calculated using a standard curve (0.1–5 µg/mL) and expressed in µg/mL.

25OH Vitamin D3 concentration was determined by ELFA (BioMerieux, Mercy-l’Etoile, France) using the Vidas-mini analyzer (BioMerieux, Mercy-l’Etoile, France). The results were automatically calculated based on the onboard calibration curve and expressed in ng/mL.

TNF-α concentration was measured using Diaclone ELISA (Diaclone SAS, Besancon, France). Absorbance was read at 450 nm with 620 nm correction. A standard curve (0–800 pg/mL) was used to calculate concentrations, expressed in pg/mL.

IL-13 concentration was assayed with Diaclone ELISA (France). Absorbance was measured at 450 nm (correction 620 nm), and concentrations were interpolated from a standard curve (0–1000 pg/mL). The results were expressed in pg/mL.

Quinolinic acid concentration was quantified using a competitive ELISA (Cloud Clone Corp., Houston, TX, USA) specific to human serum. Absorbance was read at 450 nm (correction 610 nm), and concentrations were calculated from a standard curve (0–100 ng/mL) and reported in ng/mL. IL-31 concentration was determined using Diaclone ELISA (France). Absorbance was measured at 450 nm with 620 nm correction. Concentrations were calculated from a standard curve (0–1000 pg/mL) and expressed in pg/mL.

IL-17 concentration was quantified with Diaclone ELISA (France), with absorbance read at 450 nm (correction 620 nm). Concentrations were based on a standard curve (0–1000 pg/mL) and expressed in pg/mL.

Serotonin concentration was measured using the IBL ELISA kit (Immunodiagnostik AG, Bensheim, Germany) specific to human serum. Absorbance was read at 450 nm with 620 nm correction. Concentrations were calculated from a standard curve (0–750 ng/mL) and expressed in ng/mL.

Kynurenic acid concentration was determined using a competitive ELISA kit (Cloud Clone Corp., Houston, TX, USA) dedicated to human serum. Absorbance was read at 450 nm (correction 620 nm). Concentrations were calculated from a standard curve (0–2000 ng/mL) and expressed in ng/mL.

### 2.4. Statistical Analysis

Statistical analysis was performed using Statistica 13 software (Statsoft, Tulsa, AZ, USA) and GraphPad Prism 10 (GraphPad, LaJolla, CA, USA). Continuous variables were presented using mean and standard deviation. The distribution of the variables was assessed using the Shapiro–Wilk test. To compare differences between groups, the Student *t*-test or the Wilcoxon signed rank test were used. To study the exact effects of irradiation and eliminate temporal variables, the results of measurements over time were subtracted (valueafter − valuebefore) and a Δvalue was created. The correlation analysis was performed using the Spearman correlation coefficient. A correlation coefficient ranging from 0.00 to 0.19 was considered as very weak, 0.20 to 0.39 as weak, 0.40 to 0.59 as moderate, 0.60 to 0.79 as strong, and 0.80 to 1.0 as very strong. A *p* value below 0.05 was deemed significant.

## 3. Results

**Figure 1:**
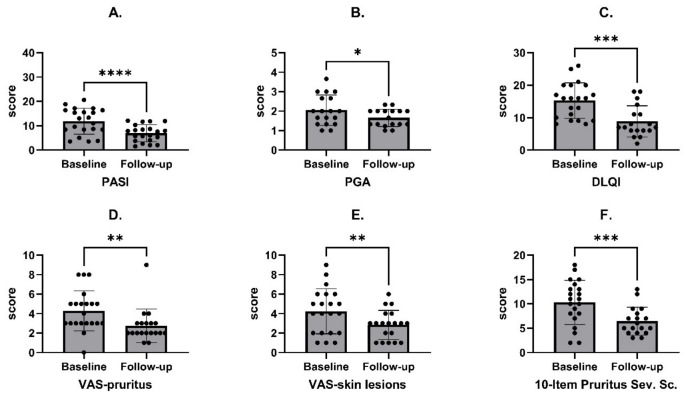
Changes in the clinical evaluation scales during the observation: (**A**) PASI, (**B**) PGA, (**C**) DLQI, (**D**) VAS-pruritus, (**E**) VAS-skin lesions, (**F**) 10-Item Pruritus Severity Scale. PASI—Psoriasis Area and Severity Index; PGA—Physician Global Assessment; DLQI—Dermatology Life Quality Index,; VAS—Visual Analog Scale. Data presented as means with standard deviations. Asterisks above the figures indicate the level of statistical significance, with *p* < 0.05 denoted by *, *p* < 0.01 by **, *p* < 0.001 by ***, and *p* < 0.0001 by ****.

Clinical evaluation demonstrated statistically significant improvement across all assessed parameters between baseline and follow-up (Figure 1, Table 2). PASI decreased significantly from a mean of 11.87 ± 5.32 at baseline to 6.90 ± 3.46 at follow-up (*p* = 0.0001), reflecting a marked reduction in lesion severity. Similarly, PGA showed a significant improvement from 2.05 ± 0.78 to 1.66 ± 0.45 (*p* = 0.0202). Patient-reported outcomes also indicated substantial clinical benefit associated with blue light irradiations. DLQI improved from 15.24 ± 5.47 to 8.83 ± 4.81 (*p* = 0.0008). Pruritus intensity, measured by both a VAS (4.29 ± 2.05 to 2.74 ± 1.73; *p* = 0.0072) and the 10-Item Pruritus Severity Scale (10.29 ± 4.54 to 6.44 ± 2.87; *p* = 0.0006), showed statistically significant reductions. Additionally, the VAS for skin lesions improved from 4.24 ± 2.32 to 2.84 ± 1.50 (*p* = 0.0110), aligning with clinician-assessed improvements. No statistically significant changes were observed in basic cardiovascular parameters between baseline and follow-up (Table 3). Systolic blood pressure (SBP) did not change significantly between baseline and follow-up, with mean values of 129.48 ± 19.01 mmHg and 125.43 ± 13.93 mmHg, respectively (*p* = 0.8213). Diastolic blood pressure (DBP) also remained stable, decreasing slightly from 77.52 ± 13.40 mmHg to 77.29 ± 8.41 mmHg (*p* = 0.8757). Similarly, heart rate (HR) showed no significant difference over time, with values of 80.14 ± 12.51 beats per minute at baseline and 79.05 ± 8.96 at follow-up (*p* = 0.7113).

**Figure 2:**
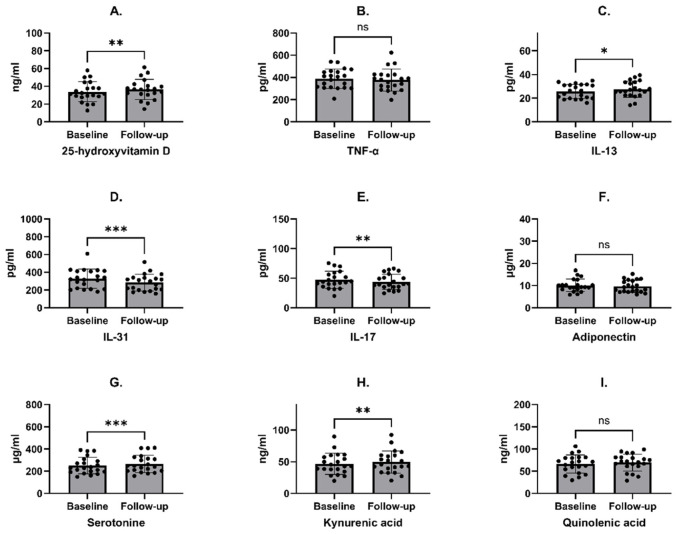
Changes in biochemical parameters during the observation. (**A**) 25-hydroxyvitamin D, (**B**) TNF-α, (**C**) IL-13, (**D**) IL-31, (**E**) IL-17, (**F**) Adiponectin, (**G**) Serotonin, (**H**) Kynurenic acid, (**I**) Quinolinic acid. Data presented as means with standard deviations. Asterisks above the figures indicate the level of statistical significance, with *p* < 0.05 denoted by *, *p* < 0.01 by **, and *p* < 0.001 by ***.

Further analysis revealed significant changes in several biomarkers between baseline and follow-up. Serum 25-hydroxyvitamin D3 levels increased from 33.84 ± 11.32 ng/mL at baseline to 36.48 ± 11.35 ng/mL at follow-up (*p* = 0.0044). Among inflammatory markers, IL-13 increased from 25.67 ± 6.01 pg/mL to 27.54 ± 6.82 pg/mL (*p* = 0.0228), whereas IL-31 decreased from 328.21 ± 106.98 pg/mL to 284.78 ± 93.71 pg/mL (*p* = 0.0007). IL-17 also showed a modest but significant reduction, decreasing from 47.30 ± 14.40 pg/mL to 43.78 ± 12.84 pg/mL (*p* = 0.0064). TNF-α decreased slightly from 388.59 ± 86.84 pg/mL to 376.51 ± 98.73 pg/mL, although this change was not statistically significant (*p* = 0.0987). Regarding neuromodulatory and metabolic markers, serotonin increased from 251.20 ± 74.67 ng/mL to 266.43 ± 78.21 ng/mL (*p* = 0.0009). Kynurenic acid content also increased significantly, increasing from 46.60 ± 16.62 ng/mL to 49.55 ± 17.59 ng/mL (*p* = 0.0106). Quinolinic acid content showed a small increase, increasing from 66.42 ± 20.20 ng/mL to 69.62 ± 18.93 ng/mL, but the difference was not statistically significant (*p* = 0.0735). Adiponectin levels remained stable (10.09 ± 2.84 µg/mL vs. 9.69 ± 2.72 µg/mL; *p* = 0.1924). Detailed results are presented in Table 4 and Figure 2.

Correlation analyses were performed to assess associations between changes in clinical parameters and alterations in biochemical markers between baseline and follow-up. Overall, most correlations were weak and did not reach statistical significance. For disease severity, a moderate positive correlation was observed between ΔPASI and ΔIL-13 (R = 0.5106, *p* = 0.0180), which was the only statistically significant association in this group. Correlations between ΔPASI and other markers, including Δ25-OH D3 (R = 0.2981, *p* = 0.1893), ΔKYNA (R = 0.2865, *p* = 0.2081), and ΔQA (R = 0.1715, *p* = 0.4573), were not significant. No significant relationships were found between ΔPGA or ΔDLQI and the analyzed biochemical parameters. For pruritus-related outcomes, ΔVAS pruritus showed a moderate positive correlation with ΔKYNA (R = 0.4296, *p* = 0.0664), although this did not reach statistical significance. Similarly, ΔVAS skin lesions tended to correlate with ΔIL-17 (R = 0.4024, *p* = 0.0876) and inversely correlate with Δ5-HT (R = −0.3318, *p* = 0.1653), but these associations were also non-significant. Correlations involving the 10-Item Pruritus Severity Scale were generally weak and not statistically significant. Detailed results are presented in Table 5.

## 4. Discussion

Blue light therapy is an emerging modality in dermatology, increasingly investigated for its potential benefits in managing chronic inflammatory skin diseases such as psoriasis [4]. While its safety profile and localized anti-inflammatory effects make it a promising alternative or adjunct to conventional phototherapies, the exact biological mechanisms underlying its therapeutic action remain incompletely understood [11]. In this study, we aimed to explore systemic biochemical and immunological responses to blue light exposure in patients with psoriasis, focusing on inflammatory and metabolic markers, as well as clinical improvement.

Contrary to our expectations, serum 25-hydroxyvitamin D3 levels increased significantly after treatment. Due to the fact that blue light lacks the UVB spectrum required for cutaneous vitamin D synthesis [12], its rise seems to not be associated with direct photochemical effect [14]. Instead, the observed increase may have been influenced by external confounders, including seasonal variation, sunlight exposure, dietary intake, or vitamin D supplementation, which were not fully controlled in the present study. Importantly, changes in vitamin D were not significantly correlated with clinical improvement, suggesting that its increase was not a primary driver of disease severity reduction in this cohort.

The cytokine response to blue light therapy showed a somewhat heterogenic pattern rather than an unvaried anti-inflammatory effect. IL-13 concentrations increased significantly after treatment, and IL-31 and IL-17 levels decreased, while TNF-α showed a non-significant downward trend. This mixed profile suggests the selective immunomodulation of specific inflammatory pathways, thus contrasting with the current literature showing that blue light therapy exerts anti-inflammatory effects through the downregulation of proinflammatory mediators [4,15,16]. Moreover, a moderate positive association between changes in IL-13 and changes in PASI (R = 0.51, *p* = 0.02) was observed, indicating that IL-13 changes may be linked to clinical response. Given the predominantly Th17/IL-23-driven pathogenesis of psoriasis, we hypothesize that IL-13 may reflect broader immunological changes accompanying clinical improvement rather than a direct pathogenical mechanism. One possible explanation for the increase in IL-13 may involve blue light-mediated regulation of the epidermal circadian rhythm. The epidermis has its own peripheral circadian clock, and keratinocytes express opsins involved in light detection [17]. In particular, peropsin has been shown to detect near-ultraviolet and blue light and to participate in light-induced circadian rhythm regulation in differentiated keratinocytes. Experimental data from skin organotypic cultures suggest that blue light may entrain epidermal circadian rhythm through peropsin and that IL-13 expression in keratinocytes may follow a circadian pattern under light–dark conditions [17]. These findings provide a plausible mechanistic link between blue light exposure and the observed increase in IL-13 in our study. In contrast, changes in IL-31, IL-17, and TNF-α were not significantly related to clinical improvement. This interpretation seems to be supported by a study performed by Buhl et al. [18], in which full-body blue light irradiation in patients with atopic dermatitis reduced itch and was associated with moderate decreases in IL-31, but did not significantly improve objective measures of atopic dermatitis severity [18]. Thus, IL-31 reduction may mainly reflect an antipruritic effect rather than sufficient suppression of the inflammatory process in atopic dermatitis. Additionally, atopic dermatitis is mainly associated with barrier dysfunction and Th2-skewed inflammation, while psoriasis, as mentioned before, is driven predominantly by keratinocyte hyperproliferation and the IL-23/Th17 axis. Recent evidence also suggests the occurrence of increased IL-31 expression in psoriatic lesions compared with healthy skin, although without a clear association with disease severity or itch intensity [19]. Therefore, the beneficial effects of blue light in psoriasis may involve mechanisms beyond IL-31 modulation.

The significant rise in serotonin levels is consistent with the known effects of light therapy on mood and circadian rhythms [20] and aligns with our previous findings [21]. While psoriasis is associated with an increased prevalence of depression, an increase in serotonin levels could contribute to an improved sense of well-being and quality of life, independent of direct skin effects [22]. The skin also has an intrinsic serotonergic system that modulates both inflammatory responses and cellular proliferation [23]. Nonetheless, its exact function in the pathogenesis of psoriasis remains unclear.

The kynurenine pathway is increasingly recognized for its involvement in immune regulation and inflammatory processes [24,25]. In this context, the significant increase in kynurenic acid, an antagonist of N-methyl-D-aspartate (NMDA) receptors, is particularly worth noting. Kynurenic acid exhibits neuroprotective and anti-inflammatory properties and its elevation may be associated with a compensatory anti-inflammatory mechanism or reflect blue light–induced alterations in tryptophan metabolism, in contrast to the findings of our previous research [21]. Interestingly, the concurrent but non-significant increase in quinolinic acid (NMDA receptor agonist with proinflammatory effects), alongside the significant rise in kynurenic acid, may indicate a change toward an anti-inflammatory effect within the kynurenine pathway, which could be favorable in the context of psoriasis, where systemic inflammation is associated with disease severity [24,26].

Adiponectin is known for its anti-inflammatory and insulin-sensitizing properties, and its levels are often altered in psoriasis and associated comorbidities [27]. The lack of significant change might indicate that the metabolic effects of blue light, if any occur, are not strongly mediated through adiponectin modulation in this patient cohort or study duration.

Lastly, while the improvements in PASI and DLQI observed in our study were statistically significant, their clinical relevance should be interpreted cautiously and in relation to established therapeutic benchmarks. In real-world studies of systemic biologic therapies, substantially higher levels of skin clearance are usually achieved [28]; therefore, blue light therapy should not be considered comparable to modern systemic treatments in patients requiring high-level disease control.

This study has several limitations. Most importantly, the single-arm pre–post design without a control group prevents causal inference; thus, the observed changes may also reflect regression to the mean, a placebo effect, or fluctuations in disease activity. In addition, the small sample size limits statistical power. Therefore, the obtained results should be interpreted with caution.

## 5. Conclusions

In summary, using blue light therapy in patients with psoriasis induced measurable systemic changes in several biochemical pathways. Serotonin, vitamin D, and kynurenic acid levels increased significantly, while cytokine responses were heterogeneous, with an increase in IL-13 and reductions in IL-31 and IL-17. Most biochemical alterations were not significantly associated with clinical outcomes, suggesting that therapeutic effects are likely mediated by multifactorial mechanisms rather than single biomarkers. Further studies are needed to clarify the biological relevance of these findings and optimize the clinical use of blue light therapy in psoriasis.

## Figures and Tables

**Table 1:** The baseline characteristics of the study group.

Variable	Whole Group (N = 21)	Males (N = 8)	Females (N = 13)
Age (years)	38.00 ± 16.98	39.75 ± 16.44	36.92 ± 17.88
Weight (kg)	69.19 ± 16.91	78.25 ± 15.13	62.00 ± 15.30
Height (cm)	168.62 ± 13.49	174.88 ± 13.26	164.77 ± 12.59
Fitzpatrick Skin Phototype	II-8, III-13	II-1, III-7	II-7, III-6

**Table 2:** Changes in the clinical evaluation scales during the observation. PASI—Psoriasis Area and Severity Index; PGA—Physician Global Assessment; DLQI—Dermatology Life Quality Index; VAS—Visual Analog Scale.

Variable	Baseline	Follow-Up	*p*-Value
PASI	11.87 ± 5.32	6.90 ± 3.46	0.0001
PGA	2.05 ± 0.78	1.66 ± 0.45	0.0202
DLQI	15.24 ± 5.47	8.83 ± 4.81	0.0008
VAS—pruritus	4.29 ± 2.05	2.74 ± 1.73	0.0072
VAS—skin lesions	4.24 ± 2.32	2.84 ± 1.50	0.0110
10-Item Pruritus Severity Scale	10.29 ± 4.54	6.44 ± 2.87	0.0006

**Table 3:** Changes in the basic cardiovascular parameters during the observation. SBP—systolic blood pressure; DBP—diastolic blood pressure; HR—heart rate.

Variable	Baseline	Follow-Up	*p*-Value
SBP (mmHg)	129.48 ± 19.01	125.43 ± 13.93	0.8213
DBP (mmHg)	77.52 ± 13.40	77.29 ± 8.41	0.8757
HR (/min)	80.14 ± 12.51	79.05 ± 8.96	0.7113

**Table 4:** Changes in biochemical parameters during the observation.

Variable	Baseline	Follow-Up	*p*-Value
25-hydroxyvitamin D3 (ng/mL)	33.84 ± 11.32	36.48 ± 11.35	0.0044
TNF-α (pg/mL)	388.59 ± 86.84	376.51 ± 98.73	0.0987
IL-13 (pg/mL)	25.67 ± 6.01	27.54 ± 6.82	0.0228
IL-31 (pg/mL)	328.21 ± 106.98	284.78 ± 93.71	0.0007
IL-17 (pg/mL)	47.30 ± 14.40	43.78 ± 12.84	0.0064
Adiponectin (µg/mL)	10.09 ± 2.84	9.69 ± 2.72	0.1924
Serotonin (ng/mL)	251.20 ± 74.67	266.43 ± 78.21	0.0009
Kynurenic acid (ng/mL)	46.60 ± 16.62	49.55 ± 17.59	0.0106
Quinolinic acid (ng/mL)	66.42 ± 20.20	69.62 ± 18.93	0.0735

**Table 5:** Correlations between the changes in clinical scores and biochemical parameters. The abbreviations have the same meaning as described earlier.

Variable		Δ25-OH D3	ΔTNF-α	ΔIL-13	ΔIL-31	ΔIL-17	ΔAPN	Δ5-HT	ΔKYNA	ΔQA
ΔPASI	R	0.2981	0.0896	0.5106	0.0624	0.0669	−0.1643	0.0136	0.2865	0.1715
	*p*-value	0.1893	0.6992	0.0180	0.7883	0.7732	0.4766	0.9532	0.2081	0.4573
ΔPGA	R	0.2477	0.2185	0.0885	0.0583	0.1821	0.0499	−0.3663	0.3257	−0.1176
	*p*-value	0.3217	0.3837	0.7271	0.8183	0.4695	0.8440	0.1349	0.1872	0.6422
ΔDLQI	R	0.0918	0.3963	0.0866	−0.0574	0.1053	−0.1335	−0.3055	0.2169	−0.0855
	*p*-value	0.7172	0.1035	0.7327	0.8212	0.6775	0.5975	0.2176	0.3873	0.7358
ΔVAS pruritus	R	0.2496	−0.0784	−0.1266	0.1382	0.2710	0.0526	−0.2130	0.4296	−0.1640
	*p*-value	0.3028	0.7496	0.6056	0.5727	0.2618	0.8307	0.3812	0.0664	0.5023
ΔVAS skin lesions	R	0.3702	−0.1484	−0.0510	0.0769	0.4024	0.2540	−0.3318	0.3523	0.1216
	*p*-value	0.1187	0.5442	0.8358	0.7543	0.0876	0.2941	0.1653	0.1390	0.6199
Δ10-Item Pruritus Severity Scale	R	0.0456	0.2614	0.1535	−0.1452	0.1224	−0.1929	−0.3517	0.1079	−0.2521
	*p*-value	0.8573	0.2947	0.5430	0.5653	0.6285	0.4430	0.1524	0.6700	0.3129

## Data Availability

The data presented in this study are available on request from the corresponding author.
